# Chikungunya and the eye: a review

**DOI:** 10.1186/1869-5760-3-35

**Published:** 2013-02-11

**Authors:** Padmamalini Mahendradas, Kavitha Avadhani, Rohit Shetty

**Affiliations:** 1 Narayana Nethralaya Post Graduate Institute of Ophthalmology, 121/C, Chord Road, Rajaji Nagar 1st ‘R’ Block, Bangalore, 560010, India

**Keywords:** Chikungunya fever, *Chikungunya virus*, Chikungunya retinitis, Optic neuritis, Anterior uveitis, Real-time polymerase chain reaction

## Abstract

Chikungunya is a self-limited, systemic viral infection that has been a major health problem since the past few years. Ocular manifestations of the disease have become more prevalent in the recent years. Currently, there is neither a specific treatment nor vaccine available for chikungunya fever. This review highlights the current understanding on the pathogenesis, systemic changes with an emphasis on ocular findings, laboratory investigations, and prevention and treatment of this disease.

## Review

Chikungunya fever is an arthropod-borne viral (arbovirus) disease [1-3] that has become a disease of global concern following its recent resurgence. Since 2006, chikungunya fever has emerged as an important infection even in non-endemic areas where travelers returning from endemic areas have become transmitters of this disease [4,5]. Impact of chikungunya fever on socioeconomic status has also been tremendous especially in countries like India [1,6].

There have been several accounts of epidemics of fever with arthralgia resembling present day chikungunya fever dating as far back as 1824 from India [7]. However, the first official description of chikungunya was made in 1952 following an outbreak on the Makonde Plateau, along the border between Tanganyika and Mozambique [8,9]. The word ‘chikungunya’ itself is derived from the language spoken in the same area as the first official outbreak. The origin of the word can be traced to the root verb ‘kungunyala’ which means ‘to dry up or become contorted’ [9,10]. Literally, the word translates to ‘that which bends up’ in reference to the stooped posture developed due to the rheumatologic manifestations of the disease. The incapacitating arthralgias also account for its other name ‘buka-buka’ meaning ‘broken-broken’, by which it is known in the Congo region [11].

### Epidemiology

#### Global scenario

Following the report from Tanganyika in 1952 [8,9], chikungunya epidemics have been reported from several parts of the world including Africa, Asia, and elsewhere. In Southeast Asia, India, Pakistan, Sri Lanka, Myanmar, Thailand, Indonesia, the Philippines, Cambodia, Vietnam, Hong Kong, and Malaysia have documented the epidemics [1,5,12,13]. Since 2003, there have been outbreaks in the islands of the Pacific Ocean, including Madagascar, Comoros, Mayotte the Seychelles, Mauritius, and the Reunion Island (Indian Ocean) [13]. Chikungunya fever has also been documented in France, Italy, Australia, and the USA where international travelers have facilitated the introduction of the virus from endemic areas [1,14,15].

Chikungunya fever re-emerged in India after nearly 32 years in October 2005 [1,5,12]. The epidemic was confirmed by an investigation carried out in several districts of Andhra Pradesh, Karnataka, and Maharastra by the National Institute of Virology [16].

### Natural history of chikungunya fever

Chikungunya fever is characterized by explosive outbreaks of epidemics followed by periods of disappearance sometimes lasting up to several decades. This behavior has been attributed to several reasons including the susceptibility of humans and the mosquito vectors of the virus- conditions facilitating mosquito breeding result in high vector density and ability of the vector to efficiently transmit the virus [1,4,5].

#### Transmission of the virus

The natural cycle of the virus is human-mosquito-human. There is, however, evidence of the existence of epizootic cycles that may retain the virus during the interepidemic period [1,4,5,17]. During epidemics, human beings serve as the *Chikungunya virus* reservoirs; during interepidemic periods, several vertebrates, such as monkeys, rodents, and birds, have been implied as the reservoir. Vertical maternal-fetal transmission has been documented in pregnant women affected by chikungunya fever [18].Chikungunya virus infects the human cornea and can be transmitted via the corneal grafts [19].

#### The virus

The virus causing chikungunya fever is an *Alphavirus* of the family *Togaviridae* whose genome consists of a linear, positive-sense, single-stranded RNA molecule, a 60- to 70-nm diameter capsid, and a phospholipid envelope [4,5]. Three lineages with distinct genotypic and antigenic characteristics have been identified from *Chikungunya virus* isolates collected from various geographical areas. These include the West-African phylogroup, the East, Central, and Southern African phylogroup, and the Asian phylogroup [16,20,21].

##### Mutations in the Chikungunya virus genome

A mutation at residue 226 of the membrane fusion glycoprotein E1 (E1-A226V) was detected in more than 90% of *Chikungunya virus* isolates from Reunion Islands in the 2005 outbreak. This mutation is postulated to have facilitated the replication and transmission of the virus by reducing the cholesterol dependence of the virus [22,23].

#### The vector

Chikungunya fever is transmitted by the bite of mosquitoes of the genus *Aedes* in the Asian region. *Aedes aegypti* is considered to be the principal vector, and *Aedes albopictus* (Asian tiger mosquito) has also recently emerged as an important vector. *A. aegypti* breeds in stored fresh water, such as that in coolers, flower vases, water tanks, and discarded household junk items like vehicle tires, coconut shells, pots, cans, and bins in urban and semiurban environments [12,24]. Adult mosquitoes rest in cool and shady areas and bite humans during daytime.

### Molecular mechanism

The principal cell types infected by chikungunya are fibroblasts, epithelial cells, and lymphoid cells [25]. In humans, chikungunya infection causes high levels of IFN-α, suggesting strong innate immunity, along with the production of IL-4, IL-10, and IFN-γ, suggesting the engagement of the adaptive immunity. Circulating T lymphocytes showed a CD8+ T lymphocyte response in the early stages of the disease and a CD4+ T lymphocyte-mediated response in the later stages [26]. An antibody-dependent enhancement mechanism similar to that suggested for Dengue viruses [27] has also been implicated in the pathogenesis.

Interferon gamma and IL-12 levels have been observed to rise dramatically during the acute phase of chikungunya fever. The level of IL-12 returns to normalcy in patients who recover. In contrast, patients who develop chronic arthritis show persistently high IL-12 levels. Histologic examination of synovia from patients with chronic arthritis following chikungunya fever has revealed joint inflammation due to macrophages containing viral material. Metalloprotease (MMP2) also contributes to tissue damage. *Chikungunya virus* leads to apoptosis through both the intrinsic and extrinsic pathway [28].

### Clinical manifestations

#### Systemic features

Chikungunya fever is known to affect all age groups. Both males and females are equally affected. The incubation period ranges between 2 to 7 days [4,28].

*Chikungunya virus* infection is characterized by the sudden onset of high-grade fever with chills, headache, malaise, arthralgia or arthritis, vomiting, myalgia, skin rash, and low back pain (Table 1). Most cases of chikungunya fever are self limiting, with recovery as the usual outcome [30].

**Table 1:** Systemic manifestations associated with chikungunya infection

	**Manifestations**
Common	Fever
	Arthralgia
	Skin rashes
	Headache
	Back ache
	Nausea
	Vomiting
	Joint swelling
	Myalgia
	Lymphadenopathy
	Fatigue
	Anorexia
Rare	Convulsions
	Meningoencephalitis
	Fulminant hepatitis
	Acute renal failure
	Respiratory failure
	Myocarditis

Certain patients, however, experience persistent joint symptoms for weeks or months and, occasionally, years after the initial onset of illness. This polyarthropathy frequently involves the small joints of the hand, wrist, and ankles and the larger joints such as the knee and shoulder. Joints are often swollen, and asymmetric involvement may occur. Disabling acute tenosynovitis is also frequently present [4,5,30,31]. The risk of developing inflammatory polyarthritis has been found to be higher if the initial acute phase lasted longer than 3 weeks [32].

Cervical or generalized lymphadenopathy may be present. Mucocutaneous manifestations, such as morbilliform eruptions, scaling, macular erythema, intertrigo, hypermelanosis, xerosis, excoriated papules, urticaria, and petechial spots have been described in patients with chikungunya fever [33,34].

Neurological complications such as meningoencephalitis have been reported during the first Indian outbreak and the French Reunion Island outbreak [5]. Other neurological manifestations reported thus far include neuropathy, myelitis, entrapment neuropathy [35], altered mental functions, seizures, focal neurological deficit (with abnormal computed tomography scan of the head and altered cerebrospinal fluid biochemistry) [36], myeloneuropathy [37], and acute flaccid paralysis [38].

Hemorrhagic manifestations, though not as common as in dengue fever, may present as epistaxis, bleeding from the gums, positive Hess test, subconjunctival bleed, and petechial rash [29]. Chikungunya and dengue infections can overlap in clinical presentation, but low platelet count in dengue can be used to differentiate between the two conditions [39]. Transient vascular disorders (e.g., Raynaud syndrome) have also been reported in relation with the occurrence of mixed cryoglobulinemia in chikungunya infection [40].

Severe systemic disease such as multiple organ involvements [41], sudden sensorineural hearing loss [42], hypokalemic periodic paralysis [43], liver failure, respiratory failure [44], and renal failure [45] are extremely rare but have been reported. A study from a pediatric intensive care unit in the Reunion Island reported encephalopathy, myocarditis, hemodynamic disorders, and even deaths due to circulatory failure following massive hemorrhage and post-infectious encephalitis in chikungunya fever [46].

#### Ocular manifestations

*Chikungunya virus* is known to affect the eye in myriad ways ranging from conjunctivitis to retinitis and even optic neuritis (Table 2). Photophobia and retro-orbital pain are often seen in the acute phase of chikungunya fever without any other signs of ocular involvement [14,47].

**Table 2:** Ocular features in chikungunya infection

**Features**	**Reference**
Conjunctivitis	[48]
Episcleritis	[45]
Non-granulomatous anterior uveitis	[45,49]
Granulomatous anterior uveitis	[45,49]
Keratitis	[49]
Retinitis with vitritis	[45,49,73]
Bilateral neuroretinitis	[49,51,52]
Multifocal choroiditis	[49,50]
Optic neuritis	[51,53,54]
Retrobulbar neuritis	[49,53,54]
Exudative retinal detachment	[45,49]
Panuveitis	[49]

Conjunctivitis that mimics other viral conjunctivitis and resolves within a week has been reported [48]. It may, however, be that this condition has been underreported or even gone unnoticed owing to its benign and self-limiting nature.

**Figure 1:**
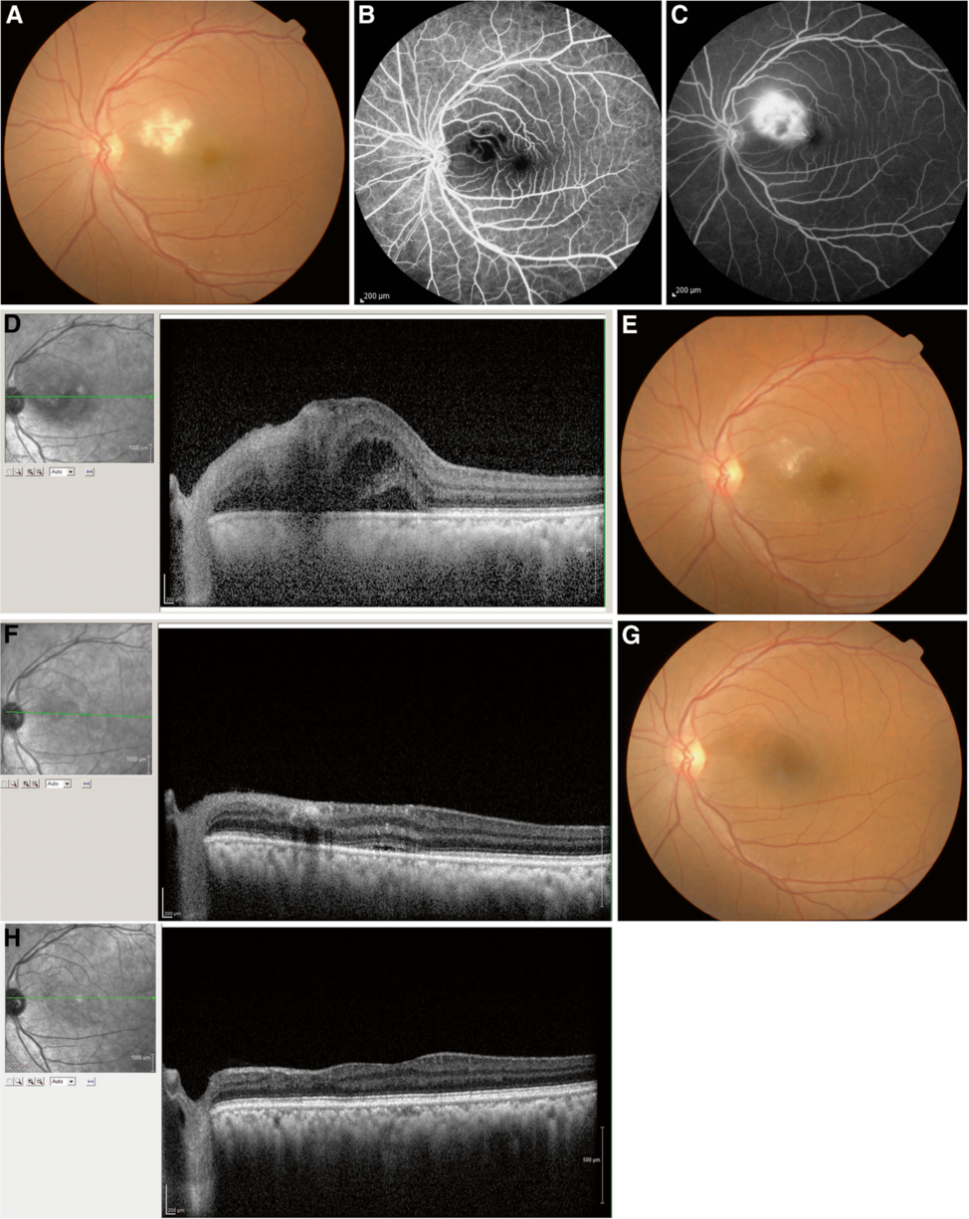
**Slit lamp anterior segment photographs of both eyes.** From a 45-year-old woman who presented with complaints of discomfort and photophobia 6 weeks following the resolution of chikungunya fever. The photograph shows pigmented keratic precipitates in the inferior cornea right eye (**A**) and pigmented and stellate keratic precipitates in the left eye (**B**), with 1+ cells and 2+ flare in the anterior chamber of both eyes. The patient received topical corticosteroids with cycloplegics and antiglaucoma therapy.

Anterior uveitis is by far the commonest presentation with both non-granulomatous [45] and granulomatous [49] variants being reported. Both variants have been noted to have pigmented keratic precipitates (Figure 1). Confocal microscopy performed in the non-granulomatous anterior uveitis has shown an infiltrative and dendritic pattern of keratic precipitates [45]. The clinical picture is similar to that seen in other viral anterior uveitis with diffuse fine keratic precipitates and anterior chamber reaction. The uveitis may be bilateral and is often associated with raised intraocular pressures [45]. The raised intraocular pressure responds well to topical antiglaucoma medications along with the topical steroids and cycloplegic agent used to treat the inflammation. The anterior segment inflammation itself takes anywhere between a few weeks to a few months to resolve. Corneal involvement in the form of viral keratitis and lagophthalmos with exposure keratitis has been described [49].

**Figure 2:**
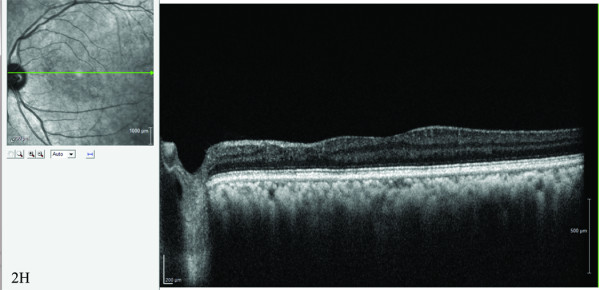
**Ocular involvements in chikungunya infection.** (**A**) Fundus photograph of the left eye showing confluent area of retinal whitening suggestive of retinitis. Fundus fluorescein angiography reveals (**B)** early hypofluorescence in the posterior pole and (**C**) late hyperfluorescence in the posterior pole. (**D**) Spectral domain optical coherence tomography (SD OCT) revealed increased reflectivity in the nerve fiber layer zone corresponding to the areas of retinitis with after shadowing and fluid-filled spaces in the outer retina with serous retinal detachment. (**E**) Fundus photograph showing resolving retinitis lesion 2 weeks after initiation of systemic steroid therapy. (**F**) SD OCT showing decreased area of hyper reflectivity in the inner retina with resolving retinal detachment. (**G**) Fundus photograph after 4 months, showing complete resolution of retinitis. (**H**) SD OCT showing resolution of retinitis with thinning of the inner retinal layers nasal to the fovea.

Posterior segment involvement may manifest as retinitis [45,48,49], choroiditis [49,50], neuroretinitis [51,52], and optic neuritis [53,54]. Retinitis may present with mild vitritis, retinal hemorrhages, retinal edema, and associated retinal vessel involvement especially in the posterior pole. Chikungunya retinitis may morphologically mimic herpetic viral retinitis; the history of fever, joint pains, and skin rash before the onset of visual symptoms is then helpful in the diagnosis of chikungunya infection [45]. Fundus fluorescein angiography reveals early hypofluorescence with late hyperfluorescence (Figure 2) corresponding to the areas of retinitis [45], vascular leakage, and/or capillary non-perfusion [30]. Optical coherence tomography shows the areas of retinitis as hyper-reflective with after shadowing, while the associated serous retinal detachment is seen as a hyporeflective area [45]. The retinitis seen in chikungunya fever may also resemble that seen in West Nile virus infection. However, clinical features such as the presence of peripheral fundus lesions and linear chorioretinal streaks are seen in West Nile virus infections and not in chikungunya retinitis [55]. This, along with serological tests for the viruses, can help differentiate the two [56].

The majority of patients with chikungunya retinitis recover well (with a good visual outcome) over a 10- to 12-week period, with only subtle retinal pigment epithelial changes remaining to suggest an earlier infection. Macular ischemia and optic disk changes account for the poor visual outcome seen in a minority of these patients [30]. Acute-onset vision loss may occur in those with optic neuritis (papillitis, retrobulbar neuritis, or neuroretinitis) secondary to *Chikungunya virus* infection.

Prompt visual recovery is usually the norm with immediate administration of systemic steroid therapy [46,53,54]. Among the optic neuritis patients, 36% of cases had simultaneous systemic and ocular manifestations, suggesting direct involvement of the virus [53]. Other neurological signs reported are bilateral external ophthalmoplegia, incongruous homonymous hemianopias [54] suggestive of optic tract lesions, and upper motor neuron facial palsy. Some other rare ocular manifestations that have been reported include exudative retinal detachment and central retinal artery occlusion [49].

#### Pathogenesis

The systemic manifestations of the fever are related to viremia, while joint involvement is believed to be an immune-mediated reaction to the viral antigen [28]. The exact mechanism of ocular involvement following chikungunya infection is not yet studied in detail. Simultaneous occurrence of systemic and ocular disease suggests the possibility of direct viral involvement such as conjunctivitis, anterior uveitis, viral retinitis, and optic neuritis. Chikungunya virus antigens were detected in keratocytes of the corneal stroma and sclera, in fibroblasts of the iris stroma and in fibroblasts of ciliary bodies suggest direct ocular involvement [19]. Late involvement of ocular tissue suggests a delayed immune response in cases of episcleritis, viral retinitis, panuveitis, and optic neuritis [53]. Antigenic mimicry between *Chikungunya virus* antigens and normal or altered host tissue proteins, immediate hypersensitivity reactions, and stimulations of a pathogenic lymphocytic reaction may be responsible for this delayed immune response [29,30].

### Laboratory diagnosis

Laboratory tests used to diagnose chikungunya infection include virus isolation, serological tests, and molecular techniques. Virus isolation and real-time polymerase chain reaction (RT-PCR) are useful during the initial viremic phase of the illness, whereas antibody demonstration from the serum is of use in the later phase of the disease.

Viral culture is the gold standard test for the diagnosis of chikungunya fever, and it is based on inoculation of mosquito cell cultures or mammalian cell cultures [4,5,28]. Specific detection of *Chikungunya virus* can be performed using RT-PCR by amplifying a fragment of the *E-2* gene [57,58]. Combined detection and genotyping of *Chikungunya virus* by targeting the *nsP1* and *E1* genes has been developed by Hasebe et al. [59]. Another specific and sensitive tool for diagnosing chikungunya infection is the one-step TaqMan RT-PCR assay that quantifies viral load in both clinical samples and cell culture. This can be used as an indicator of active infection [60]. RT-PCR with real-time loop-mediated isothermal amplification has also been found to be a useful method for rapid diagnosis [61].

Enzyme-linked immunosorbent assay (ELISA), immunochromatographic test (ICT), indirect immunofluorescent method, hemagglutination inhibition, or neutralization techniques may be used to detect chikungunya IgM and IgG antibodies in the serum. The IgM antibodies are detectable after a mean period of 2 days from the onset of the fever, and they persist for several weeks, sometimes even up to 3 months, whereas IgG antibodies can be detected in the convalescent samples and persist for years [62]. The sensitivity and specificity of the ICT ranged from 1.9% to 3.9% and 92.5% to 95.0%, respectively, in a study by Blacksell et al., suggesting poor diagnostic accuracy of the commercial antibody-based assays for the diagnosis of acute chikungunya infections [63].

Blood counts may be normal, or patients may have leukopenia with relative lymphocytosis. Erythrocyte sedimentation rate is significantly elevated, and low rise in C-reactive protein is positive in acute cases [62].

Nearly all the studies reporting ocular manifestations of chikungunya fever have utilized either virus detection from the serum using RT-PCR or antibody detection from the serum of patients. Ocular fluid (aqueous humor) analysis for *Chikungunya virus* using RT-PCR was reported to be positive in a case of Fuchs' heterochromic iridocyclitis [64] and another case of Fuchs' viral anterior uveitis. [65] RT-PCR showed 88.5% sensitivity, 100% specificity, 100% positive predictive value, and 97.5% negative predictive value, suggesting that the diagnostic accuracy of RT-PCR assays is clinically acceptable for the diagnosis of acute chikungunya infection [63]. Unfortunately, the availability of the RT-PCR methodology is limited in low-resource settings where this disease is commonly found. There is, thus, an urgent need to design and evaluate simple chikungunya RNA- or antigen-based detection assays, such as loop-amplified [66-68] or antigen-capture ELISA [69] technologies.

### Management

The *Chikungunya virus*, an RNA virus, is unstable over time, and there is no specific antiviral drug available to date for the treatment of *Chikungunya virus* infection. Treatment at the acute stage of the disease is symptomatic with antipyretics (acetaminophen is preferred over aspirin so as to avoid bleeding complications) and nonsteroidal anti-inflammatory drugs [12,70]. Chronic arthritis due to chikungunya infection has been variously treated with chloroquine phosphate [68,71], corticosteroids, disease-modifying antirheumatic drugs, and even tumor necrosis factor blockers [72].

Anterior uveitis has been treated with topical steroids and cycloplegic agents [47,49]. Associated ocular hypertension has been managed with topical beta blockers and oral or topical carbonic anhydrase inhibitors. Systemic steroids are used for the control of inflammation in posterior uveitis, panuveitis, and optic neuritis [45,46,48-53,73]. A few cases of chikungunya fever with ocular manifestations involving the posterior segment have been empirically treated with acyclovir and systemic steroids although the efficacy of acyclovir has been doubtful [45,73]. The search for a specific antiviral drug against the *Chikungunya virus* is still on.

#### Prevention

In the absence of a specific treatment or vaccines against *Chikungunya virus*, prevention of disease spread by vector control still remains the most important mode of control for chikungunya infection. Elimination of breeding sites and source reduction are effective methods. The vector *A. aegypti* is a container habitat species and breeds primarily in artificial containers and receptacles. Measures to control mosquito breeding include covering of water tanks, cisterns, and other water storage equipment; removal of tires and coconut shells that may collect water; regular emptying of bird baths and pet water bowls; trimming of tall grass/weeds; and the introduction of larvivorous fish such as the guppy in ornamental water tanks [74].

Persons living in or traveling to mosquito-infested areas must use protective measures to prevent mosquito bites. These maybe in the form of insecticide-treated nets, use of mosquito repellant creams (such as those containing *NN*-diethyl-*m*-toluamide) on the exposed skin, and even insecticide spraying to kill the mosquitoes [74].

Another strategy in its developmental stages is specific immunoprophylaxis with human anti-*Chikungunya virus* immunoglobulins. Human polyvalent immunoglobulins have been purified from plasma samples obtained from donors in the convalescent phase of chikungunya infection, and the preventive and curative effects of these immunoglobulins have been investigated in mouse models of *Chikungunya virus* infection [75]. This may constitute a safe and efficacious prevention strategy for individuals exposed to *Chikungunya virus* and are at risk of severe infection, such as neonates born to viremic mothers [76] and adults with underlying systemic conditions.

To this day, there is no commercial vaccine for chikungunya. Live chikungunya vaccines have been abandoned to avoid the possible risk of viral persistence [77].

## Conclusions

Ocular manifestations of chikungunya infection can be present at the time of fever or may manifest after many weeks. Anterior uveitis, optic neuritis, and retinitis are the commonest manifestations. Diagnosis of chikungunya can be made either by RT-PCR from the serum or ocular fluids or by demonstration of antibodies in the serum. In the absence of a specific antiviral regimen, the treatment of ocular disease is supportive. Exact pathogenesis of the ocular manifestations, development of specific antiviral therapy, and vaccination against chikungunya are fields that require further research.

## Consent

Written informed consent was obtained from the patient for publication.

## Competing interests

The authors declare that they have no competing interests.

## Authors’ contributions

PM designed the article outline, collected the data, and drafted the manuscript. KA collected the data and drafted the manuscript. RS prepared the manuscript. All authors read and approved the final manuscript.
